# Physicochemical aspects of adsorption efficiency of nanocrystalline ceria toward antibiotics, herbicides, and inorganic phosphates

**DOI:** 10.1039/d5ra05301c

**Published:** 2025-10-14

**Authors:** Jakub Ederer, Luboš Vrtoch, Petr Ryšánek, Matouš Bárta, Viktorie Neubertová, Zdeňka Kolská

**Affiliations:** a Faculty of Environment, Jan Evangelista Purkyně University Pasterurova 3632/15 400 96 Ústí nad Labem Czech Republic jakub.ederer@ujep.cz +420-475-284-170 +420-475-284-111; b Faculty of Science, Jan Evangelista Purkyně University Pasteurova 3632/15 Ústí nad Labem 400 96 Czech Republic

## Abstract

Nanoceria oxides (ceria, CeO_2_) have emerged as promising materials for the effective adsorption of various pollutants, including antibiotics, herbicides, and inorganic phosphates, owing to their unique physicochemical properties. This study explores the adsorption efficiencies of nanoceria samples synthesized using various laboratory procedures. The adsorption behavior of cephalexin (CEF), 2,4-dichlorophenoxyacetic acid (2,4D), and inorganic phosphate (IP) was evaluated using the Freundlich, Langmuir, and Langmuir–Freundlich isotherm models. The results showed that the adsorption of IP, 2,4D, and CEF followed the Freundlich and Langmuir–Freundlich isotherms with maximum adsorption capacities of 68.6 mg g^−1^ (IP), 65.9 mg g^−1^ (CEF), and 83.4 mg g^−1^ (2,4D), respectively. IP, 2,4D, and CEF adsorption on ceria samples follow pseudo-second-order kinetics, suggesting that the adsorption rate relies on the availability of adsorption sites and is controlled by chemisorption. The specific interaction of pollutants with ceria samples was evaluated by FTIR. The calculated Gibbs free energy (Δ*G*°) values indicate that the adsorption of pollutants onto the ceria materials is a spontaneous process. Among the tested samples, Ce-PER and Ce-AMN demonstrated superior adsorption capacity due to their high surface area. These results highlight the promise of ceria materials as highly versatile and effective adsorbents for removing various pollutants in the water treatment process.

## Abstract 2

Adsorption study of nanoceria (CeO_2_) for cephalexin, 2,4D, and phosphate: isotherm/kinetic modeling, FTIR, and thermodynamics confirm spontaneous process; Ce-PER and Ce-AMN show superior performance in adsorption capacity.

## Graphical abstract

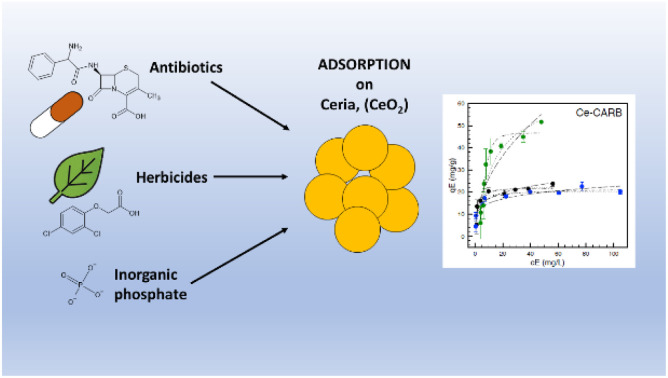

## 1. Introduction

The increasing contamination of water by antibiotics, herbicides, and inorganic phosphates has become a significant environmental challenge due to their persistence, toxicity, and potential ecological problems. Compounds such as 2,4-dichlorophenoxyacetic acid (2,4D), cephalexin (CEF), and inorganic phosphate (IP) are frequently detected in wastewater and natural water bodies due to their widespread use in agriculture and healthcare.^1^ Their persistence and potential toxicity have prompted research into novel adsorbents that can efficiently capture these contaminants. Antibiotics contribute to the spread of antibiotic-resistant bacteria,^2^ while herbicides disrupt ecological balance and biodiversity.^3^ Inorganic phosphates, on the other hand, lead to eutrophication, resulting in algal blooms and oxygen depletion in aquatic systems.^4^ These pollutants cannot be completely removed by conventional water treatment methods such as chemical precipitation, coagulation, and filtration; therefore, the search for innovative materials and technologies capable of solving this problem is being developed.^5,6^ Nanomaterials offer a promising solution to the limitations of traditional water treatment systems. Due to their high surface area, tunable surface chemistry, and unique catalytic properties, nanomaterials exhibit exceptional adsorption capacities.^7–9^ Among various nanomaterials studied for environmental remediation, cerium oxide (CeO_2_, ceria) has attracted significant attention due to its unique physicochemical properties, such as high surface area, tunable redox activity, and exceptional catalytic behavior.^10,11^ Ceria is well known for its facile switchability between the Ce^3+^ and Ce^4+^ valence states,^12^ promoting the high mobility of lattice oxygen and extending ceria from an ordinary adsorbent to a catalyst/reactive adsorbent^13^ and making ceria a potentially highly effective adsorbent/catalyst for removing various hazardous contaminants from water.^5,14,15^ Nanoceria offers significant advantages over other adsorbents and nanomaterials due to its unique redox properties, high oxygen storage capacity, and regenerative antioxidant behavior.

A wide variety of materials have been studied for removing 2,4D, CEF and IP, including magnetite/ceria composites,^16^ palygorskite,^17^ zeolite/MnO_2_ nanoparticles,^18^ and Al_2_O_3_@Fe_2_O_3_ (ref. 19) or other materials,^4,20,21^ demonstrating diverse removal capacities and adsorption properties for CEF, 2,4D and IP depending on their composition and structural properties. Unlike the previously mentioned nanomaterials, which often suffer from insufficient adsorption capacity, complex synthesis (*e.g.*, composite materials), or high economic costs, nanoceria exhibits a strong affinity for various contaminants, including heavy metals and organic pollutants^22–24^ while maintaining excellent stability under different environmental conditions.

Their prolonged reusability and enhanced efficiency of ceria in pollutant removal and catalysis further increase their attractiveness. Furthermore, their biocompatibility and low toxicity make them superior to other metal-based nanomaterials, making them highly suitable for applications in water purification,^15^ biomedical applications,^25^ and environmental remediation.^5,26^

**Fig. 1:**
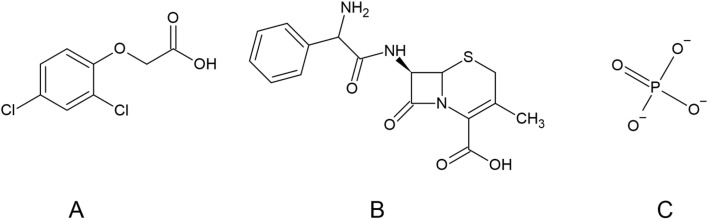
Structure of 2,4D (A), CEF (B), and IP (C).

2,4D (Fig. 1A) is a widely used herbicide that targets broadleaf weeds in agricultural and residential environments. While highly effective, it poses environmental risks due to its potential to contaminate soil, water, and air through runoff, spray drift, and leaching.^27,28^ The World Health Organization (WHO) classifies 2,4D as moderately hazardous (class II) and limits its concentration in drinking water to 70 μg L^−1^ to protect human and animal health.^28^ Runoff from treated areas can pollute nearby water bodies, harming aquatic ecosystems by affecting non-target species and causing bioaccumulation in the food chain, as well as groundwater contamination.^29,30^ Long-term human exposure has been linked to endocrine disruption and increased cancer risk, underscoring the importance of careful management.^3,31^ Similarly, CEF (Fig. 1B), a first-generation cephalosporin antibiotic used for treating bacterial infections,^17,32^ poses environmental challenges when inadequately removed by wastewater treatment plants.^33^ Residual CEF entering natural waters poses a threat to aquatic life and promotes antimicrobial resistance, a significant global health concern.^34,35^ IP (Fig. 1C), commonly found as phosphate salts such as calcium and sodium phosphate, is essential for biological functions, including energy transfer *via* ATP, nucleic acid formation, and bone health.^4^ Environmentally, phosphate acts as a vital nutrient for plant growth, but excessive runoff from fertilizers and detergents leads to eutrophication, harmful algal blooms, oxygen depletion, and ecosystem damage.^19,36^ Together, 2,4D, CEF, and IP illustrate how chemical contamination and nutrient overload create complex environmental and health challenges. Addressing these issues requires integrated approaches involving improved chemical use, advanced wastewater treatment, pollution monitoring, and regulatory measures to balance agricultural productivity with the protection of ecosystems and public health.

In this study, a set of nanocrystalline ceria samples prepared using various synthesis methods was employed to evaluate their potential for removing 2,4D, CEF, and IP from a model water system. The mutual interaction of ceria with selected pollutants (IP) was studied using Fourier transform infrared spectroscopy (FTIR), X-ray fluorescence spectroscopy (XRF), and powder X-ray diffraction (XRD). The prepared ceria materials were characterized using advanced analytical techniques, including scanning electron microscopy (SEM), XRD, determination of surface area and porosity, dynamic light scattering (DLS), electrokinetic potential measurements, and acid–base titrations. As shown, the prepared ceria samples exhibit good adsorption capacity toward IP, CEF, and 2,4D. This research may help drive the development of eco-friendly and effective water treatment technologies, thereby contributing to a cleaner and safer environment. The present study systematically investigates the adsorption of several classes of pollutants onto nanocrystalline ceria within a model aqueous system. Our goal is to elucidate the fundamental adsorbent–pollutant interactions, thereby creating a solid foundation for future research focused on the material's performance in real water and groundwater. Our study was designed to establish a baseline understanding of the adsorbent's intrinsic capabilities, providing a solid foundation for future research. The results highlight the importance of exploring specific applications of nanoceria for pollutant removal, which is crucial for developing efficient, scalable, and environmentally friendly water treatment strategies.

## 2. Experimental

### 2.1. Chemicals

Analytical gradient-grade chemicals were used in this study. Cerium nitrate hexahydrate, cephalexin hydrate (CEF), 2,4-dichlorophenoxyacetic acid (2,4D), and ammonium bicarbonate were purchased from Sigma-Aldrich (Germany). Disodium phosphate (IP) was purchased from Lach-Ner Inc. For HPLC measurements, methanol, acetonitrile, and formic acid were used and purchased from VWR Inc. (Czech Republic). Deionized water from the GORO Pharmpur system (Goro, Prague, Czech Republic) with mixed-bed ion exchange purification was used. The Spectroquant® Phosphate test purchased from Merck Millipore (Germany) was used for the determination of free inorganic phosphate.

### 2.2. Ceria preparation

Nanocrystalline ceria was prepared using wet chemical methods with cerium(iii) nitrate hexahydrate and deionized water, as described elsewhere.^37^ Samples were labeled by synthesis method: Ce-AMN (ammonium hydroxide precipitation), Ce-CARB (cerium carbonate precipitation and annealing), Ce-HMT (hexamethylenetetramine precipitation), Ce-PER (reflux of peroxo-complexes), and Ce-UREA (urea precipitation and annealing).

### 2.3. Characterization of samples

The FTIR spectra were obtained using VERTEX 70v Infrared spectrometer (Bruker, Germany) in diffuse reflectance mode (DRIFT) within the 4000–400 cm^−1^ wavenumber range with 64 scans per spectrum and a 4 cm^−1^ resolution. Raw FTIR data were processed by OPUS software (v. 8.7). The obtained data were further processed using Microsoft Excel 2021, OriginPro 2024, and Plot v2.

X-ray diffraction analysis (XRD) was performed on the Panalytical X'Pert PRO diffractometer in symmetrical reflection mode (Cu Kα = 1.5418 Å radiation, 40 kV, 30 mA) and the X'Celerator 1-dimensional detector.

Changes in the chemical composition of the samples were analyzed using a wave-dispersive X-ray fluorescence spectrometer (XRF) Rigaku Primus IV with SQX software and a standardless method of fundamental parameters. This method allows to measure the concentration of elements in the range F–U in concentration from ppm to 100%. The relative error of measurement is approximately 5%. Samples were analysed in the form of pressed tablets.

To determine the sample-specific surface area (SSA) and pore volume, nitrogen adsorption/desorption isotherms were used. Samples were degassed at 50 °C for 24 hours. Afterwards, 66-points adsorption and desorption isotherms were recorded with nitrogen (99.999%, Linde) at liquid nitrogen temperature using an Anton Paar Instrument NOVA 3200e. Surface morphology was analyzed using a scanning electron microscope SU5000 (Hitachi, Japan). See SI for more details.

### 2.4. Surface acid–base characterization and zeta potential determinations

The slightly modified acid–base titration method published elsewhere^38^ was used to evaluate the number of surface hydroxyl groups and pH(PZC) using an automatic titrator (794 Basic Titrino, Metrohm, Switzerland) with potentiometric endpoint determination.

Zeta potential and particle size distribution of the synthesized ceria materials were analyzed using the Litesizer™ 500 (Anton Paar, Austria). Electrophoretic light scattering (ELS) was applied to determine the zeta potential, and dynamic light scattering (DLS) was employed to determine the hydrodynamic diameter and size distribution of the ceria nanoparticle suspensions. The system was integrated with a Metrohm automatic titrator featuring an 867 pH module and 846 dosing interface, all operated *via* Kalliope™ software. See SI for more details.

### 2.5. Batch adsorption experiment

Adsorption studies were realized in batch experiment mode using a 100 mL Pyrex bottle with 50 mg of ceria sample and 50 mL stock solution of 2,4D, CEF, or IP (concentration ranges: 10–100 mg L^−1^ for IP and 2,4D, 5–125 mg L^−1^ for CEF). The experiment was carried out for 3 hours (IP) and 24 hours (CEF and 2,4D) at 25 ± 1 °C to ensure that equilibrium was reached. The equilibration time was chosen based on our previous experience and adsorption kinetic measurements.^16,39^ The free IP concentration was determined using a commercially available phosphate kit and the ammonium molybdate spectrophotometric method at 880 nm. The concentration of free CEF and 2,4D were measured using HPLC, and the concentration of IP/CEF/2,4D was calculated using a previously obtained calibration curve. The adsorbed amount of selected pollutant on the ceria samples at equilibrium *q*_E_ (mg g^−1^) was calculated using the following equation (eqn (1)):1
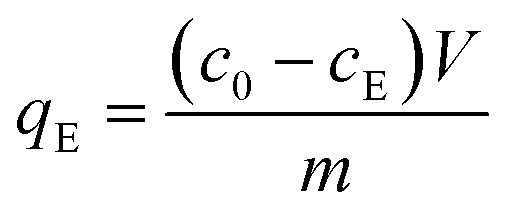
where *c*_0_ and *c*_E_ (mg L^−1^) are the initial and equilibrium concentrations, *V* (L) is the initial volume of the pollutant solution and *m* (g) is the mass of the ceria used in the experiment.

### 2.6. Adsorption kinetics

To study adsorption kinetics, the Pyrex bottle (100 mL) was used along with 100 mg of ceria samples. In all experiments, a freshly prepared stock solution of pollutants (IP, CEF, 2,4D) was used with an initial concentration of 100 mg L^−1^ and a volume of 100 mL. At predetermined intervals for IP (5, 10, 15, 30, 60, 120, and 180 min) and CEF/2,4D (30, 40, 60, 120, 180, 240, 300, 360 and 1440 min), 1.5 mL aliquots were collected into Eppendorf vials (2 mL) and centrifuged (4 min/6000 rpm). The concentration of free IP was measured spectrophotometrically at 880 nm using a commercial phosphate kit. The concentration of free CEF and 2,4D were measured using HPLC. These concentrations were assessed based on a previously established calibration curve. The amount of adsorbed IP/CEF/2,4D at time *q*_*t*_ (mg g^−1^) was calculated using eqn (2).2
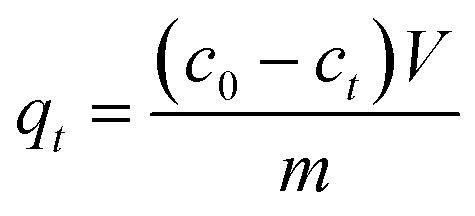
where *c*_0_ and *c*_*t*_ (mg L^−1^) are the initial and equilibrium concentrations in time *t* (min), *V* (L) is the initial volume of the phosphate solution, and *m* (g) is the mass of the ceria sample used in the kinetics experiment.

### 2.7. Analytical methods

The HPLC analysis of CEF and 2,4D was realized using the LaChrom HPLC system (Merck Hitachi) consisting of a L-7100 pump, L-7400 variable wavelength UV/vis detector operating at 262 nm (CEF) and 230 nm (2,4D), and a Rheodyne 7725i injection valve with a 20 μL sampling loop was used. CEF HPLC analysis was realized on Arion® Polar C18 column (100 × 4.6 mm, 5 μm) in the isocratic elution mode with the 15 mM NaH_2_PO_4_ pH = 3.3/methanol (65/35) as the mobile phase (1.0 mL min^−1^). The HPLC analysis of 2,4D was performed in isocratic elution mode with acetonitrile/water (50/50) as the mobile phase (1.0 mL min^−1^, water contains 0.1% HCOOH), and the SIELC Newcrom A column (150 × 4.6 mm, 5 μm) was used.

## 3. Results and discussion

### 3.1. Characterization of samples

**Fig. 2:**
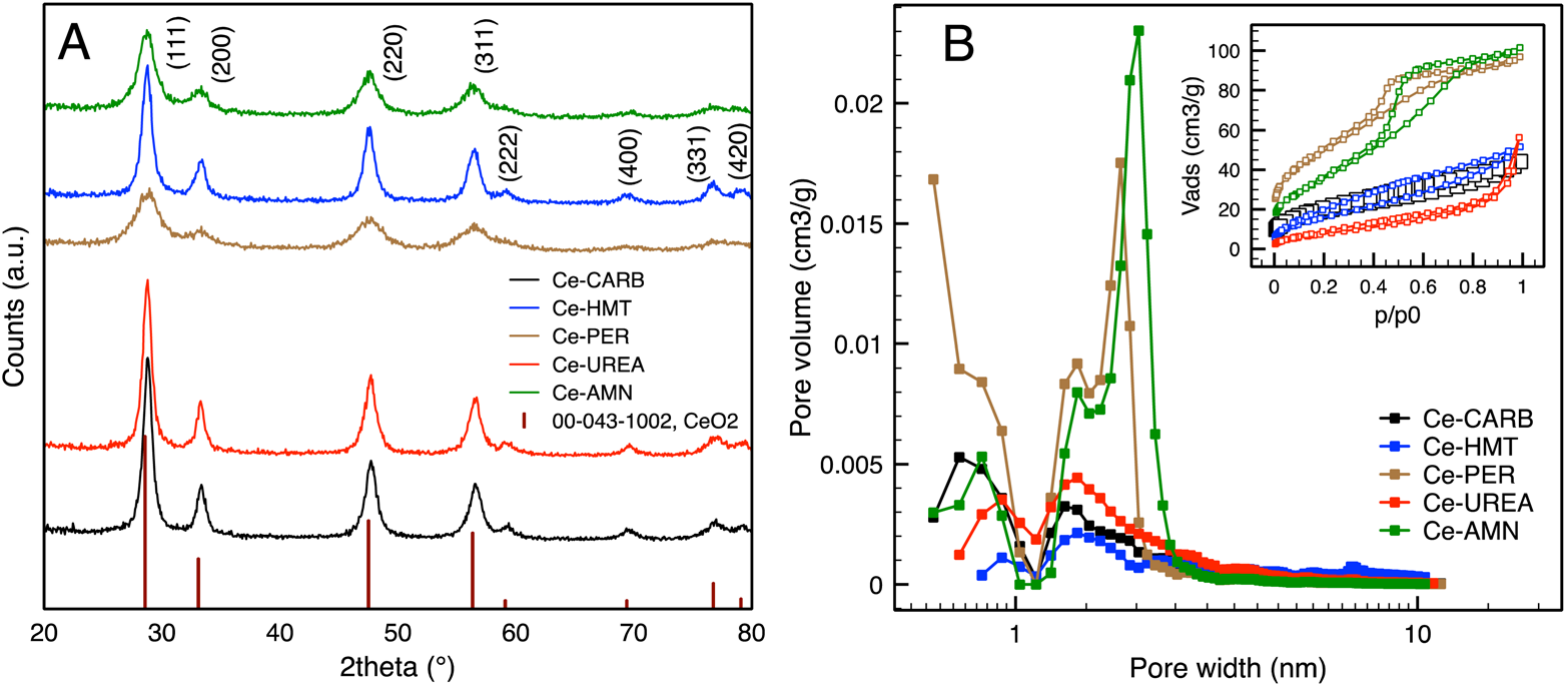
XRD patterns of ceria samples (A) and pore size distribution obtained from DFT analysis and the nitrogen-desorption BET isotherms of ceria samples (B).

The N_2_ adsorption/desorption isotherms and pore size distribution are presented in Fig. 2B and Table 1. The nitrogen adsorption/desorption isotherms exhibit a typical type IV behavior, characteristic of porous and mesoporous materials with relatively small particle size (Fig. 2B). These isotherms exhibit hysteresis loops classified by IUPAC (1985) as types H4 and H2,^40^ which is typical for microporous and mesoporous materials. The results align well with those published in ref. 37. It is evident that the annealing/drying temperature and synthesis procedure significantly influence the specific surface area (Table 1). The X-ray diffraction (XRD) patterns of the prepared samples are shown in Fig. 2A. A reduction in diffraction line sharpness is observed for Ce-HMT, Ce-AMN, and Ce-PER samples. All diffraction lines correspond to the characteristic face-centered cubic fluorite-type structure, with peaks assigned to the (111), (200), (220), (311), (222), (400), (331), and (420) planes, located at 28.761°, 33.281°, 47.748°, 56.561°, 59.157°, 69.594°, 76.729°, and 79.108°, respectively (ICDD PDF 34-0394). The average cubic crystallite size (CCS), ranging from 3 to 14 nm, was calculated from the broadening of diffraction lines using Scherrer's analysis. The calculated CCS aligns well with previously reported data for similar ceria materials.^25,37^

**Table 1:** The specific surface area (SSA) and total pore volume (*V*_pore_) of prepared samples obtained from nitrogen-desorption using BET and DFT analysis. Mean cubic crystallite size of ceria samples calculated from XRD data

Sample	SSA (m^2^ g^−1^)	*V*_pore_ (cm^3^ g^−1^)	Cubic crystallite size^a^ (nm)
Ce-CARB	69.8 ± 1.4	0.062 ± 0.001	13.5
Ce-UREA	60.6 ± 1.5	0.072 ± 0.001	11.6
Ce-PER	179.7 ± 0.6	0.136 ± 0.001	3.1
Ce-HMT	30.0 ± 1.4	0.076 ± 0.004	8.7
Ce-AMN	132.4 ± 5.4	0.142 ± 0.003	4.6

a The average deviation is ± 2.5 nm.

The nanostructure morphology of the prepared ceria samples was studied by SEM (Fig. S1). Ce-UREA exhibits flake-like particles assembled into bigger agglomerates without uniform shape. Ce-CARB exhibits plate-like aggregates formed by hexagonal-shaped particles. Ce-HMT, Ce-PER, and Ce-AMN show irregular aggregates with a random distribution of size and shape, consisting of very small primary nanoparticles (as confirmed by XRD).

The chemical composition of ceria obtained by XRF measurements is summarized in Table S2.

The XRF and XRD (Fig. S5) analyses confirmed the presence of P-containing moieties on the ceria after IP adsorption. The phosphorus content in the ceria samples increased from an initial 0.0% to approximately 1.2 wt%. The complete elemental composition, as determined by XRF, is summarized in Table S2. Some other elements (*e.g.*, F, SiO_2_) were found only in minor amounts (see detailed results in Table S2).

### 3.2. Ceria surface acid–base characteristic

The number of surface hydroxyl groups (*q*_OH_) was evaluated from titration curves, while the pH(PZC) was obtained from TOTH curves, representing the total concentration of protons consumed in the titration process. Additional information can be found elsewhere,^38^ and the calculated data are summarized in Table 2, Fig. S2A and B. Zeta potential determinations were used to evaluate the surface charge and stability of ceria samples in aqueous solution. Fig. S3A shows the ceria sample's zeta potential as a function of pH, and the calculated data are listed in Table 2. The isoelectric point (IEP) of ceria samples was evaluated from the plot in Fig. S3A, and the values are summarized in Table 2. The hydrodynamic particle size distribution of ceria samples measured using dynamic light scattering (DLS) is presented in Fig. S3B. Table 2 presents the average particle size (diameter) and the polydispersity index (PDI). A PDI value below 0.30 indicates a uniform particle size distribution and good suspension stability of the ceria samples in water at their native pH.^41^ See SI for more details.

**Table 2:** pH of isoelectric point (IEP) and average particle size, polydispersity index (PDI) calculated from DLS and the number of surface hydroxyl groups calculated from titration curves and pH(PZC) for the prepared ceria samples

Sample	pH (IEP)	Average particle size from DLS ± SE (nm)	PDI ± SE	*q*_OH_ ± SE (mmol g^−1^)	pH(PZC)
Ce-CARB	6.12	1667 ± 46	29.9 ± 1.4	0.190 + 0.007	4.6
Ce-UREA	6.87	2021 ± 121	27.4 ± 0.7	0.198 + 0.012	4.6
Ce-PER	9.40	754 ± 21	28.1 ± 1.6	0.137 + 0.010	4.5
Ce-HMT	8.21	1084 ± 44	24.4 ± 0.4	0.402 + 0.011	8.1
Ce-AMN	5.21	1261 ± 69	21.4 ± 1.2	0.112 + 0.004	4.9

The nature and behavior of ceria in aqueous solutions is related to pH(PZC), which is the determining parameter for identifying the surface charge. From the pH(PZC) value, it can be determined whether the ceria surface will be positively or negatively charged at a given pH. The pH(PZC) remains similar, except for the Ce-HMT sample. The Ce-HMT higher pH(PZC) value could be associated with the remaining HMT residues. The calculated number of hydroxyl groups and pH(PZC) values nicely correlated with data published elsewhere.^37^

### 3.3. Adsorption isotherm data

The adsorption isotherms were used to describe 2,4D, CEF, and IP adsorption. The most widely used isotherm models, Freundlich (F), Langmuir (L), and Langmuir–Freundlich (LF) mathematical models were used in this work. Freundlich (eqn (3)), Langmuir (eqn (4)), and Langmuir–Freundlich (eqn (5)) isotherm models in non-linear form can be expressed by eqn (3)–(5):^42^

3 *q*_E_ = *K*_F_ × *c*_E_^1/*n*_F_^

**Fig. 3:**
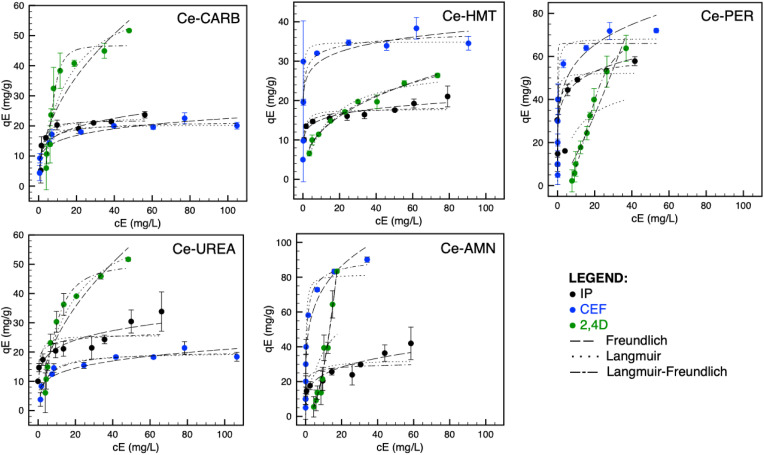
Adsorption isotherms: Freundlich (––), Langmuir (⋯), and Langmuir–Freundlich (–-–) for IP, CEF, and 2,4D on prepared ceria samples.

4
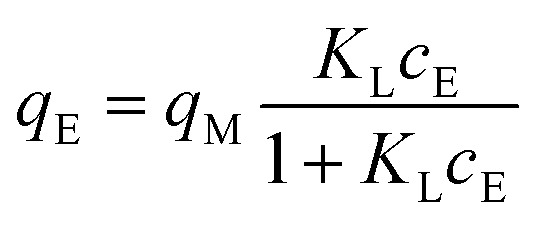
5
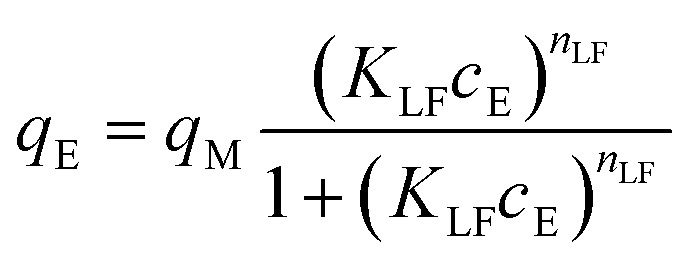
where *q*_E_ is the equilibrium amount of given pollutant adsorbed per unit weight of ceria (mg g^−1^), *q*_M_ is the maximum adsorption capacity (mg g^−1^), *K*_F_, *K*_L_ and *K*_LF_ are the Freundlich ((mg g^−1^) (mg L^−1^)^1/*n*_F_^), Langmuir (L mg^−1^) and Langmuir–Freundlich (L mg^−1^) adsorption constants, respectively; *n*_F_ is the adsorption intensity, and *n*_LF_ is the heterogeneity parameter. The extrapolated experimental data with Freundlich, Langmuir and Langmuir–Freundlich mathematical models are presented in Fig. 3, and the data are summarized in Tables 3–5.

**Table 3:** Freundlich, Langmuir, Langmuir–Freundlich model constants and correlation coefficients for the adsorption of IP by nanoceria

Sample	Freundlich			Langmuir			Langmuir–Freundlich			
	*K*_F_ (mg g^−1^) (mg L^−1^)^1/*n*_F_^	*n*_F_	*R* ^2^	*K*_L_ (L mg^−1^)	*q*_M_ (mg g^−1^)	*R* ^2^	*K*_LF_ (L mg^−1^)	*q*_M_ (mg g^−1^)	*n*_LF_	*R* ^2^
Ce-CARB ± SE	10.5	4.89	0.7786	0.56	22.8	0.8669	0.61	21.8	1.35	0.8729
	1.73	1.24		0.17	1.36		0.16	1.71	0.65	
Ce-UREA ± SE	14.1	5.56	0.8154	1.67	25.4	0.4410	4.0^a^	29.9	0.34	0.5640
	1.86	1.22		1.29	2.33		0.0	12.4	0.55	
Ce-PER ± SE	33.7	6.63	0.9127	5.92	52.3	0.9157	1.60	68.6	0.35	0.9351
	2.93	1.28		2.51	3.11		3.89	23.2	0.18	
Ce-HMT ± SE	11.5	8.24	0.9030	1.86	17.8	0.7288	4.0^a^	20.3	0.36	0.7954
	0.64	1.12		0.66	0.75		0.0	4.92	0.36	
Ce-AMN ± SE	13.8	4.15	0.8808	0.57	32.5	0.5657	4.0^a^	32.8	0.41	0.6161
	2.02	0.76		0.48	4.07		0.0	7.30	0.37	

a Fixed parameter.

**Table 4:** Freundlich, Langmuir, Langmuir–Freundlich model constants and correlation coefficients for the adsorption of CEF by nanoceria

Sample	Freundlich			Langmuir			Langmuir–Freundlich			
	*K*_F_ (mg g^−1^) (mg L^−1^)^1/*n*_F_^	*n*_F_	*R* ^2^	*K*_L_ (L mg^−1^)	*q*_M_ (mg g^−1^)	*R* ^2^	*K*_LF_ (L mg^−1^)	*q*_M_ (mg g^−1^)	*n*_LF_	*R* ^2^
Ce-CARB ± SE	9.81	5.56	0.8517	0.71	20.4	0.9263	0.56	21.9	0.73	0.9406
	1.28	1.08		0.19	0.82		0.23	1.90	0.20	
Ce-UREA ± SE	7.33	4.40	0.8578	0.27	19.8	0.9320	0.25	20.6	0.86	0.9348
	1.18	0.83		0.06	0.90		0.09	2.09	0.26	
Ce-PER ± SE	36.3	5.10	0.8459	4.28	68.3	0.9244	6.91	65.9	2.63	0.9608
	4.41	1.01		1.12	4.10		0.70	2.99	0.70	
Ce-HMT ± SE	23.9	9.94	0.6346	6.13	34.9	0.6225	4.14	40.7	0.36	0.6512
	2.90	3.47		2.93	2.91		16.1	27.1	0.68	
Ce-AMN ± SE	43.7	4.39	0.9301	3.53	81.7	0.9642	1.64	94.3	0.62	0.9845
	3.64	0.58		0.78	3.74		0.70	7.58	0.10	

**Table 5:** Freundlich, Langmuir, Langmuir–Freundlich model constants and correlation coefficients for the adsorption of 2,4D by nanoceria

Sample	Freundlich			Langmuir			Langmuir–Freundlich			
	*K*_F_ (mg g^−1^) (mg L^−1^)^1/*n*_F_^	*n*_F_	*R* ^2^	*K*_L_ (L mg^−1^)	*q*_M_ (mg g^−1^)	*R* ^2^	*K*_LF_ (L mg^−1^)	*q*_M_ (mg g^−1^)	*n*_LF_	*R* ^2^
Ce-CARB ± SE	8.77	2.11	0.7849	0.07	67.7	0.8579	0.15	46.7	3.19	0.9545
	2.87	0.46		0.03	11.1		0.01	2.60	0.74	
Ce-UREA ± SE	7.70	1.96	0.8834	0.06	70.5	0.9427	0.12	50.5	1.87	0.9761
	1.97	0.31		0.02	8.02		0.01	3.21	0.31	
Ce-PER ± SE	0.84	0.81	0.9226	0.10^a^	50.6	0.4559	0.05	70.6	3.17	0.9944
	0.44	0.11		0.0	8.63		0.002	3.65	0.28	
Ce-HMT ± SE	4.92	2.54	0.9823	0.07	29.2	0.9566	4.4 × 10^−3^	72.8	0.52	0.9843
	0.42	0.15		0.01	1.81		0.01	62.3	0.14	
Ce-AMN ± SE	0.29	0.50	0.9686	0.10^a^	74.3	0.4445	2.8 × 10^−2^	463.5^c^ (83.4^b^)	2.21	0.9691
	0.14	0.05		0.0	13.7		5.3 × 10^−2^	1370.9^c^	0.77	

a Fixed parameter.

b Experimentally measured value.

c Data obtained by non-linear regression using OriginPro 2024.

The data obtained by non-linear fitting are summarized in Tables 3–5, along with the maximum values of *q*_M_ for each individual sample. The data suggested that the LF isotherms well-fitted to measure data for CEF and 2,4D (Tables 4 and 5), which nicely correlated with information published elsewhere.^16^ In contrast, the IP adsorption (Table 3) can be well-described by F (Ce-UREA, Ce-HMT, and Ce-AMN) and LF (Ce-CARB and Ce-PER) isotherm models considering *R*^2^ under the concentration range studied.

The heterogeneity parameter (*n*_F_) is used to classify the adsorption process as chemical (*n*_F_ < 1), physical (*n*_F_ > 1), or linear (*n*_F_ = 1). A value of 1/*n*_F_ < 1 or 1/*n*_F_ > 1 indicates normal adsorption, whereas other values suggest cooperative adsorption. The measured values of *n*_F_ > 1 and 1/*n*_F_ < 1 confirm that the adsorption process is predominantly physical and that the Freundlich isotherm model is favorable for Ce-UREA, Ce-HMT, and Ce-AMN samples in the adsorption of IP.

Using the *R*_L_ parameter from the Langmuir isotherm, the favorable parameter *K*_L_*c*_0_ was calculated (eqn (6))6
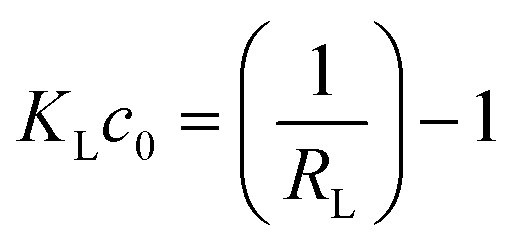


The values of *K*_L_*c*_0_ can be categorized into five intervals, each corresponding to distinct adsorption isotherm shapes as described in ref. 43. When *K*_L_*c*_0_ = 0 isotherm is linear. For *K*_L_*c*_0_ values between 0.1 and 1, the isotherm is considered pseudo-linear, and between 1 and 10, the isotherm is considered favorable, while 10 < *K*_L_*c*_0_ < 100 is classified as highly favorable, and 100 < *K*_L_*c*_0_ < 1000 is pseudo-irreversible.^43^ The calculated *K*_L_*c*_0_ values (data not shown) for 2,4D and all ceria samples indicate a favorable and pseudo-linear isotherm. However, for IP and CEF, the *K*_L_*c*_0_ values ranged from 1 to 770, suggesting favorable, highly favorable, and pseudo-irreversible isotherms, respectively.^43^

The Langmuir constant *K*_L_ was used to calculate the separation factor *R*_L_, and the data are presented in SI (see Fig. S4). The *R*_L_ value indicates the nature of the adsorption to be either irreversible if *R*_L_ = 0, favorable if 0 < *R*_L_ < 1, linear if *R*_L_ = 1, or unfavorable if *R*_L_ > 1.^43^ The *R*_L_ value below 1 indicates that the adsorption process becomes more favorable with increasing CEF, 2,4D, and IP concentration, confirming the high ceria affinity towards selected pollutants.

The data summarized in Table 6 compare the adsorption capacities for 2,4D, CEF, and IP across various materials, including those prepared and used in this study. The data for selected pollutants exhibited comparable or partly lower adsorption capacities relative to other materials. The higher *q*_M_ value can be attributed to a comparable or higher BET surface area or to different adsorption mechanisms occurring in other samples. This is related to adsorption conducted under native conditions, *i.e.*, no pH adjustment, thereby reflecting the inherent properties of the ceria samples.

**Table 6:** Comparison of maximum adsorption capacity, experimental conditions, and mathematical models for CEF, 2,4D, and IP adsorption by cerium materials and other metal oxides/composites

Material	Pollutant	*q*_M_ (mg g^−1^)	Mathematical model^a^^,^^b^	*T* (°C)	Contact time (h)	References
Ceria (different synthesis)	CEF	20.6–94.3	LF	25	24	This work
Zeolite/MnO_2_ nanoparticles		20.9	L	25	2	18
Biochar from corn bract		13.9	L	25	48	44
Palygorskite		112.33	L	28	24	17
Magnetite/ceria composite		28.1–110.7	LF	25	24	16
Ceria (different synthesis)	2,4D	46.7–83.4	LF	25	24	This work
Magnetite/ceria composite		19.9–55.7	LF	25	24	16
Activated carbon from carbonized chest nut shell		0.93	L	35	4.2	20
Co–Al–Cl layered double hydroxide		27.2	L	25	1	21
Algal magnetic activated carbon		60.61	L	30	1	45
Ceria (different synthesis)	IP	20.3–68.6	LF	25	3	This work
CeO_2_ nanoparticles		0.3–0.4	L	NA^c^	24	36
Hydrous CeO_2_ (annealed at 60–1200 °C)		6.5–99.8	L	25	24	46
MgO(100) functionalized cellulose sponge		26.8	L	25	2	4
Al_2_O_3_@Fe_2_O_3_ composite		106.2	L	25	2	19

a L – Langmuir mathematical model.

b LF – Langmuir–Freundlich mathematical model.

c NA – not available data.

### 3.4. Adsorption mechanisms of pollutants

The F model for IP adsorption suggested non-ideal and reversible adsorption at heterogeneous surfaces. Several studies^47–49^ have linked the phosphate adsorption mechanism to the formation of insoluble CePO_4_ species (confirmed by FTIR), resulting from the reaction of Ce^3+^ ions present at defect sites within the ceria crystal lattice. As reported by Ko *et al.*,^48^ the cerium oxidation state changes from Ce^4+^ to Ce^3+^ with an increasing concentration of phosphate adsorbed on ceria, leading to the formation of CePO_4_ through the reaction between Ce^3+^ and phosphate. The XRF and XRD analyses confirmed the presence of P-containing moieties on the ceria surface, suggesting the formation of CePO_4_. Following the adsorption of IP, the phosphorus content in the ceria samples increased from an initial 0.0 mass% to approximately 1.2 mass%. The complete elemental composition, as determined by XRF, is summarized in Table S2. From the XRD pattern (see Fig. S5), the presence of crystalline CePO_4_ and hydrogen phosphate is evident.

The increased *q*_M_ for IP in certain samples is likely associated with a higher number of Ce^3+^ sites, which bind phosphate preferentially over Ce^4+^ sites.^47^ Our results show that Ce-UREA and Ce-PER (12 at% Ce^3+^) align with this observation. However, Ce-HMT sample (16 at% Ce^3+^) does not follow this trend, which may be attributed to the inaccessibility of Ce^3+^ sites, potentially hindered by the presence of –OH groups (Ce-HMT has the highest number of –OH groups; see Table 2). The elemental surface composition obtained by XPS measurement is summarized in Table S1.

IP is likely to adsorb readily onto naturally positively charged samples, whereas the adsorption of negatively charged IP anions may be suppressed (on negatively charged ceria).^50^ Conversely, the decrease in phosphate adsorption at higher pH levels is a well-documented phenomenon for sesqui(hydr)oxide adsorbents.^51^ At higher pH levels, intense competition between PO_4_^3−^ species and OH^−^ ions is likely present, leading to significant repulsion between phosphate and hydroxyl ions and thereby reducing phosphate adsorption.^19^ This reduction can be explained by (1) the conversion of surface hydroxyl groups from the highly reactive M–OH_2_^+^ form to the less reactive M–OH, where M represents a metal atom, and (2) the competitive interaction with OH^−^ ions.^51^

The higher adsorption capacities of Ce-AMN and Ce-PER for CEF and 2,4D are likely attributed to their high specific surface area to crystallite size ratio and bigger pore volume (*V*_pore_), which can be beneficial for adsorption. In contrast, Ce-HMT did not exhibit such a high adsorption capacity despite having the highest number of surface hydroxyl groups and being the only ceria with an alkaline value of pH(PZC). This suggested a partly negative effect of hydroxyl groups and the potential unavailability of Ce^3+^/Ce^4+^ sites, which may play a crucial role in the adsorption process. At alkaline pH, CEF and 2,4D exist in their anionic forms, which could hinder adsorption due to electrostatic repulsion between the negatively charged ceria surface and the anionic forms of CEF and 2,4D.

The LF model suggests that ceria behaves as a material with heterogeneous surfaces and unequal bonding sites at lower pollutant concentrations. However, at higher concentrations, the ceria surface exhibits identical and equivalent sites, with a finite number of these sites available and monolayer adsorption capacity. The *q*_M_ values for CEF increase in the following order: Ce-UREA < Ce-CARB < Ce-HMT < Ce-PER < Ce-AMN. The highest *q*_M_ observed for Ce-AMN can be attributed to its second-largest SSA (132.4 m^2^ g^−1^), pore volume (0.142 cm^3^ g^−1^), and smallest CCS (4.6 nm), all of which enhance the adsorption of both CEF and 2,4D. The primary mechanism driving CEF adsorption involves electrostatic interaction between the CEF zwitterion and positively/negatively charged –OH groups on the ceria surface. The solution pH and the surface charge of ceria play a crucial role in this process. According to Sutherland,^52^ optimal adsorption occurs when CEF exists in its zwitterionic form. A similar adsorption mechanism, *i.e.*, electrostatic interactions, will be involved for 2,4D on materials with different structures and surface characteristics.^29,31^ 2,4D adsorption on Ce-AMN (LF model, Table 5) shows an unrealistic *q*_M_ value of 1370.9 mg g^−1^ obtained by non-linear regression. The experimentally measured isotherm data do not display a plateau phase for Ce-AMN. Therefore, the experimentally obtained *q*_M_ value corresponding to the highest *q*_M_ value was added to Tables 5 and 6.

Additionally, Ce-PER, which has the most defect-rich surface,^37^ featuring Brønsted and Lewis sites, may further facilitate CEF, 2,4D and IP adsorption. The Ce-CARB, Ce-UREA, Ce-PER, and Ce-AMN exhibit comparable number of –OH groups and pH(PZC) values (see Table 2). The electrostatic interactions governing the adsorption process can be elucidated by comparing the pH(PZC) of the ceria adsorbent with the dissociation constants (p*K*_a_) of the target pollutants. The ceria surface exhibits a positive charge at a solution pH below its pH(PZC), while it becomes negatively charged at pH > pH(PZC). Conversely, the pollutants CEF (p*K*_a_ = 2.56, 6.88),^52^ 2,4D (p*K*_a_ = 2.73),^31^ and IP (p*K*_a_ = 2.0, 6.8, and 12.3)^53^ predominantly exist in anionic forms at pH > p*K*_a_. Therefore, in an acidic to neutral pH range where the pH is simultaneously above the pollutants' p*K*_a_ and below the adsorbent's pH(PZC), a favorable electrostatic attraction is established between the anionic pollutant species and the positively charged ceria surface, driving the adsorption process. Therefore, it can be assumed that the number of surface hydroxyl groups is not crucial for CEF and 2,4D adsorption. In contrast, other physicochemical parameters, such as SSA, *V*_pore_, and CCS, are likely to play a significant role in the adsorption of CEF and 2,4D. A study^38^ highlights the importance of physicochemical parameters in the adsorption of inorganic phosphates on ceria samples annealed at various temperatures.

The strong correlation of IP (Ce-UREA, Ce-HMT, and Ce-AMN) with the Freundlich model indicates that the ceria surface is heterogeneous. Adsorption occurs with multilayer coverage and involves interactions between the adsorbed phosphate molecules and already-created insoluble CePO_4_ (ref. 54) (see eqn (7)). Insoluble form of CePO_4_ is primarily formed by the Ce^3+^ ions reaction with anionic form of phosphate (PO_4_^3−^, HPO_4_^2−^, H_2_PO_4_^−^) presented in ceria defects.^48^ The proposed interaction mechanisms for IP, 2,4D, and CEF are presented in Fig. 7.

7 Ce^3+^ + PO_4_^3−^ → CePO_4_↓

The efficiency of cerium-based materials in removing phosphate from water is significantly affected by the presence of other coexisting anions, with the nature and degree of this interference depending heavily on the specific chemistry of the competing ion and the adsorbent used.^36,55^ This is particularly evident with several anions that pose a significant challenge. Silicates (SiO_3_^2−^), for example, exhibit a profound inhibitory effect due to their chemical and structural similarity to phosphate, leading to intense competition for adsorption sites and a potential removal rate decrease of up to 82.88%.^55^ Similarly, bicarbonates (HCO_3_^−^) and carbonates (CO_3_^2−^) interfere by both competing for binding sites and increasing the solution's pH to a less favorable alkaline state.^55,56^ Other chemically homologous contaminants, such as arsenate (As(v)), which has a similar ionic structure to phosphate, and fluoride (F^−^), also act as strong competitors, with some adsorbents even showing a stronger affinity for arsenate over phosphate.^56–58^ Conversely, many common simple anions show little to no negative influence, which supports the hypothesis that the primary removal mechanism is chemisorption (inner-sphere complexation) rather than weaker electrostatic interactions. Ions such as chlorides (Cl^−^), sulfates (SO_4_^2−^), and nitrates (NO_3_^−^) typically have a minimal impact; in fact, chlorides and sulfates can sometimes even slightly enhance phosphate removal.^55,56^ Ultimately, this means that while common electrolytes pose little threat, the performance of ceria-based adsorbents can be severely compromised by species like silicates, arsenates, and bicarbonates.^55^ For this reason, experimental confirmation of an adsorbent's capacity in the presence of these specific interfering ions is a crucial and necessary step before its final acceptance and deployment in real-world wastewater treatment scenarios.

### 3.5. Kinetics studies

**Fig. 4:**
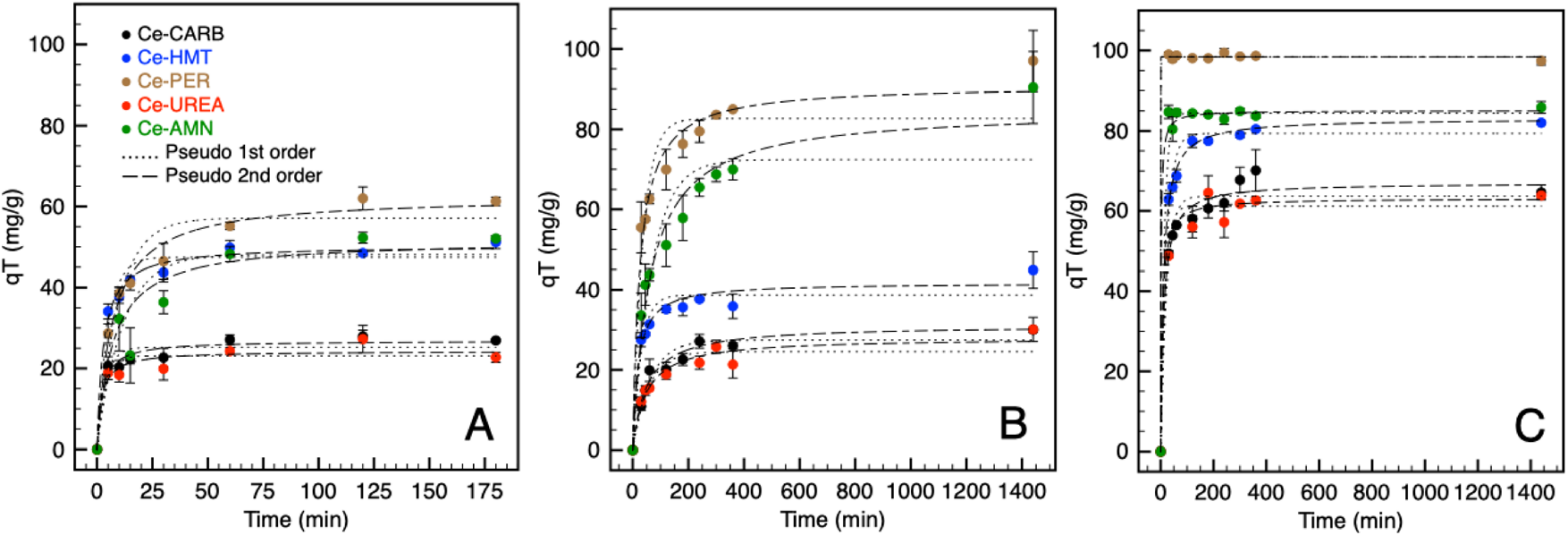
Adsorption kinetics of IP (A), CEF (B), and 2,4D (C) on prepared ceria samples.

The experimental data were fitted by the mathematical model for pseudo-first (eqn (8)) and pseudo-second (eqn (9)) order kinetics^59,60^ to evaluate the IP, CEF and 2,4D adsorption kinetics on the ceria samples. The pseudo-first-order (PFO) and pseudo-second-order (PSO) kinetic models are represented by equations (eqn (8) and (9)), with corresponding data presented in Table 7 and illustrated in Fig. 4.

8 *q*_*t*_ = *q*_E_ × (1 − e^−*k*_1_*t*^)

9
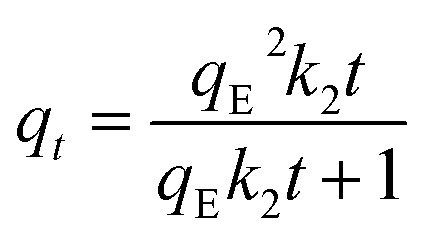
in eqn (8) and (9), *q*_E_ and *q*_*t*_ (mg g^−1^) correspond to adsorption capacity at equilibrium (can be defined by eqn (1) and (2)) and at any time *t* (min), respectively. The *k*_1_ (min^−1^), *k*_2_ (g mg^−1^ min^−1^) is the pollutant adsorption rate constant. From the data obtained from the PSO model, the approaching equilibrium factor *R*_A_ was calculated (eqn (10)), which represents the characteristic of the kinetic curves of an adsorption system.10
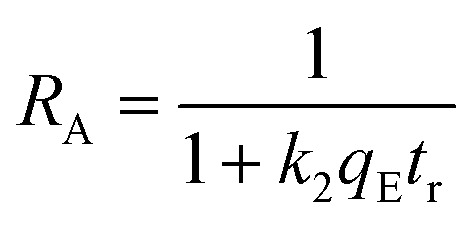
in eqn (10), *t*_r_ corresponds to the longest adsorption time of the kinetic experiment, *q*_E_ is the adsorption capacity at equilibrium (mg g^−1^), and *k*_2_ (g mg^−1^ min^−1^) is the pseudo-second-order adsorption rate constant. The adsorption curve is described as “approaching equilibrium” when 0.1 < *R*_A_ < 1, “well-approaching equilibrium” when 0.1 < *R*_A_ < 0.01, and *R*_A_ < 0.01 is “drastically approaching equilibrium”.^61^ The values of *R*_A_ indicate that the adsorption of IP and CEF is well-approaching equilibrium; in contrast, 2,4D is drastically approaching equilibrium. A larger CCS of the ceria adsorbent resulted in an increase in the *R*_A_ value, especially for 2,4D. For other tested pollutants (CEF and IP) the effect was not observed. However, *R*_A_ is influenced not only by the particle size of the adsorbent but also by the properties of solution, adsorbent, and adsorbate.^62^ In our experiments, the CCS, number of surface –OH groups, and SSA varied over the used samples, while other factors were held constant, such as adsorbent dosage, initial pollutant concentration, and temperature.

**Table 7:** The adsorption kinetics parameters for IP, CEF, and 2,4D adsorption on the ceria samples

Sample	Pollutant	Pseudo-first order			Pseudo-second order			
		*q*_E_ ± SE (mg g^−1^)	*k*_1_ ± SE × 10^−2^ (min^−1^)	*R* ^2^	*q*_E_ ± SE (mg g^−1^)	*k*_2_ ± SE × 10^−3^ (g mg^−1^ min^−1^)	*R* ^2^	*R*_A_
Ce-CARB	IP	25.2 ± 1.19	26.3 ± 7.66	0.9165	26.9 ± 1.02	16.6 ± 5.60	0.9637	1.2 × 10^−2^
Ce-HMT		47.4 ± 1.65	20.2 ± 3.60	0.9567	50.3 ± 1.05	7.10 ± 1.15	0.9892	1.5 × 10^−2^
Ce-PER		57.1 ± 2.77	10.3 ± 1.89	0.9362	62.5 ± 1.86	2.30 ± 0.37	0.9838	3.7 × 10^−2^
Ce-UREA		23.1 ± 1.41	27.4 ± 10.0	0.8931	24.3 ± 1.39	20.1 ± 10.7	0.9301	1.1 × 10^−2^
Ce-AMN		50.8 ± 3.63	5.48 ± 1.35	0.9043	56.5 ± 4.49	1.34 ± 0.52	0.9309	6.8 × 10^−2^
Ce-CARB	CEF	27.4 ± 1.18	1.60 ± 0.26	0.9365	31.2 ± 1.36	0.66 ± 0.14	0.9624	3.3 × 10^−2^
Ce-HMT		38.7 ± 1.43	3.32 ± 0.59	0.9252	41.8 ± 1.45	1.26 ± 0.31	0.9610	1.3 × 10^−2^
Ce-PER		82.7 ± 3.24	2.76 ± 0.48	0.9220	91.0 ± 2.65	0.43 ± 0.08	0.9749	1.8 × 10^−2^
Ce-UREA		24.5 ± 1.53	1.78 ± 0.40	0.8831	28.0 ± 1.57	0.76 ± 0.21	0.9433	3.2 × 10^−2^
Ce-AMN		72.4 ± 4.66	1.45 ± 0.34	0.8720	84.9 ± 4.20	0.19 ± 0.04	0.9562	4.0 × 10^−2^
Ce-CARB	2,4D	63.7 ± 1.54	4.41 ± 0.63	0.9643	67.0 ± 1.51	1.31 ± 0.28	0.9810	7.9 × 10^−3^
Ce-HMT		79.4 ± 1.27	4.40 ± 0.42	0.9842	83.1 ± 0.88	1.13 ± 0.12	0.9957	7.3 × 10^−3^
Ce-PER		98.4 ± 0.24	11.9 ± 3.18	0.9997	98.4 ± 0.34	47.5 ± 83.6	0.9996	1.5 × 10^−4^
Ce-UREA		61.2 ± 1.21	57.9 ± 0.89	0.9747	63.1 ± 1.65	2.48 ± 0.91	0.9728	4.4 × 10^−3^
Ce-AMN		84.0 ± 0.52	7.12 ± 0.68	0.9987	85.1 ± 0.66	6.61 ± 2.87	0.9981	1.2 × 10^−3^

The adsorption kinetics is a relatively fast process, according to data in Table 7 and Fig. 4. All samples exhibit rapid initial adsorption kinetics for IP, CEF, and 2,4D, with maximum adsorption achieved within 60 min for IP and 400 min for both CEF and 2,4D. With a further increase in the adsorption time, the sorption rate no longer changes and reaches adsorption equilibrium.

Table 7 shows that the *R*^2^ value is highest for the PSO model, with a value close to 1, indicating that CEF, 2,4D, and IP adsorption follow PSO kinetics. Moreover, the calculated *q*_E_ value from the PSO model closely matched the experimental *q*_E_ value for all pollutants, confirming the suitability of this model. The results align with the findings for other adsorbents, such as magnesia/ceria composite,^16^ Co–Al–Cl-layered double hydroxide,^21^ zeolite/MnO_2_ nanoparticles,^18^ corn bract biochar,^44^ MgO functionalized cellulose sponge,^4^ Al_2_O_3_/Fe_2_O_3_ composite^19^ used for removing CEF, 2,4D, or IP, respectively. For some samples and 2,4-D, it is challenging to determine which kinetic model is more appropriate, *i.e.*, PFO or PSO, for Ce-PER, Ce-UREA, and Ce-AMN, due to the close values of *R*^2^.

The PSO model, commonly used for pollutant adsorption from aqueous solutions,^63^ suggests that the adsorption of IP, CEF, and 2,4D is controlled by chemisorption. Chemisorption may serve as the rate-limiting step in the pollutant adsorption process. These results are consistent with,^16,18,45^ which reported that the adsorption of IP, 2,4D, and CEF indicates chemical adsorption. However, this model cannot fully address the overall kinetics, as it is difficult to determine the specific contributions of each individual step to the entire process. Under our experimental conditions, the adsorption rate was comparable to data elsewhere.^31,35,64^

Variations in pore volume among the ceria samples can be attributed to differences in the rate constants, especially for Ce-PER and Ce-AMN in the adsorption of 2,4D and CEF. In contrast, for Ce-CARB, Ce-UREA, and Ce-HMT, which exhibit comparable pore volumes and specific surface area, it is evident that these characteristics are not crucial for determining the adsorption rate. The faster rate constant observed for 2,4D can likely be attributed to its smaller molecule size compared to CEF, enabling it to penetrate deeper into the pores. The relatively fast adsorption can be attributed to the large surface area and pore size/volume (Ce-PER, Ce-AMN), which facilitate effective contact with 2,4D in the model solution. These properties promote the diffusion of 2,4D from the bulk solution to the active sites on the prepared materials.^47,60^

The fastest adsorption rate is likely associated with several parameters: (a) the optimal number of surface hydroxyl groups acting as ion-exchanger; (b) nanoparticles crystallite size and SSA; (c) pore volume/size; (d) oxygen vacancies and defects in the crystal lattice. In contrast, a higher adsorption rate of CEF was observed for samples with smaller pore volume. This can be attributed to the influence of mesopore volume on adsorption capacity, which is typical for larger adsorbates. Moreover, mesopores reduce the diffusion path length in micropores, which has a more significant impact on the diffusion of larger molecules.^65^ The molecular size of the organic compounds significantly influenced both the overall adsorption rate and the dominant mass transfer mechanism.^66^ The calcined samples, Ce-CARB and Ce-UREA, exhibited comparable rate constants for all pollutants except for IP on the Ce-CARB sample. The calcined samples have similar physical–chemical parameters (*i.e.*, CCS, *V*_pore_, SSA, and *q*_OH_), suggesting that these factors are unlikely to be the rate-determining step for adsorption. The adsorption rate for IP is closely related to the improvement in crystalline lattice quality along with the evolution of novel cavity-shaped defects for annealed samples.^67,68^ According to Aškrabić *et al.*,^69^ dislocations in the crystal lattice serve as the primary sites for unsaturated Ce^3+^ ions and oxygen vacancies. These are highly influenced by the annealing temperature, which can play a significant role in enhancing the adsorption process and its kinetics.^70–72^

For 2,4D, it is evident that a larger pore volume and SSA are beneficial for the sorption rate (Ce-PER, Ce-AMN). For the other samples (Ce-CARB, UREA, and HMT), which have comparable *V*_pore_ and SSA, it is clear that these two parameters are unlikely to be key factors in determining the adsorption behavior. The faster sorption process can likely be attributed to the smaller molecular size of 2,4D compared to CEF, allowing it to penetrate deeper into bigger pores more easily. For CEF, the sorption rate decreases as the pore volume increases, which could be related to the bulkier structure of the CEF molecule compared to 2,4D. A similar effect was observed for the adsorption of various types of amino compounds on the carbonaceous nanoparticles.^73^ Smaller molecules can more easily penetrate the pores; however, the sorption rate may depend on the strength of the sorbent–sorbate affinity. For the selected analytes, it is evident that chemisorption is taking place. Chemisorption is characterized by an initially rapid rate, as the number of binding sites is high and analytes quickly attach. As the number of active sites decreases (due to being already occupied), the sorption rate slows down.

### 3.6. Thermodynamic parameters

To evaluate the feasibility of adsorption and determine if the adsorption process is physisorption or chemisorption, the Gibbs free energy (Δ*G*°) was calculated using the Langmuir adsorption constant (*K*_L_) based on methodology published elsewhere.^74,75^ The Gibbs free energy can be expressed by the following equation (eqn (11)).

11 Δ*G*° = −*RT* ln *K*_L_

where Δ*G*° (kJ mol^−1^) is the change in the Gibbs free energy, *R* is the universal gas constant (8.314 J K^−1^ mol^−1^), *T* (K) is the thermodynamic temperature, and *K*_L_ is the Langmuir adsorption constant (L mol^−1^). The values are listed in Table 8; the Δ*G*° < 0 values correspond to the adsorption process being spontaneous and feasible. In contrast, Δ*G*° > 0 indicates a non-spontaneous process that requires energy input.

**Table 8:** Calculated values of Gibbs free energy using the Langmuir adsorption constant for individual samples and pollutants

Sample	Δ*G*° (kJ mol^−1^)		
	IP	2,4D	CEF
Ce-CARB	−28.20	−23.91	−30.78
Ce-UREA	−28.26	−23.53	−28.29
Ce-PER	−32.82	−24.80	−35.32
Ce-HMT	−29.12	−23.91	−36.12
Ce-AMN	−27.06	−24.80	−34.75

The Gibbs free energy change associated with physisorption typically lies within the range of 0 to −20 kJ mol^−1^. In contrast, chemisorption is characterized by significantly more negative values, generally ranging from −80 to −400 kJ mol^−1^.^76^ Negative values (Table 8) confirm that the process is spontaneous and feasible for all samples and pollutants, requiring no external energy input. The Δ*G*° values, particularly for CEF and IP, are slightly higher than those typical for physisorption, yet they remain below the threshold for chemisorption. This indicates that the adsorption of these compounds on all ceria samples is a physicochemical process, predominantly governed by physisorption. The calculated Δ*G*° for 2,4D was consistent with a physisorption process. The Δ*G*° values for IP, 2,4D, and CEF are approximately two to three times higher than those reported in previous studies,^17,31,57^ indicating that the adsorption process is more thermodynamically favorable on our materials.

### 3.7. Characterization of ceria samples before and after adsorption of IP, 2,4D and CEF by FTIR

**Fig. 5:**
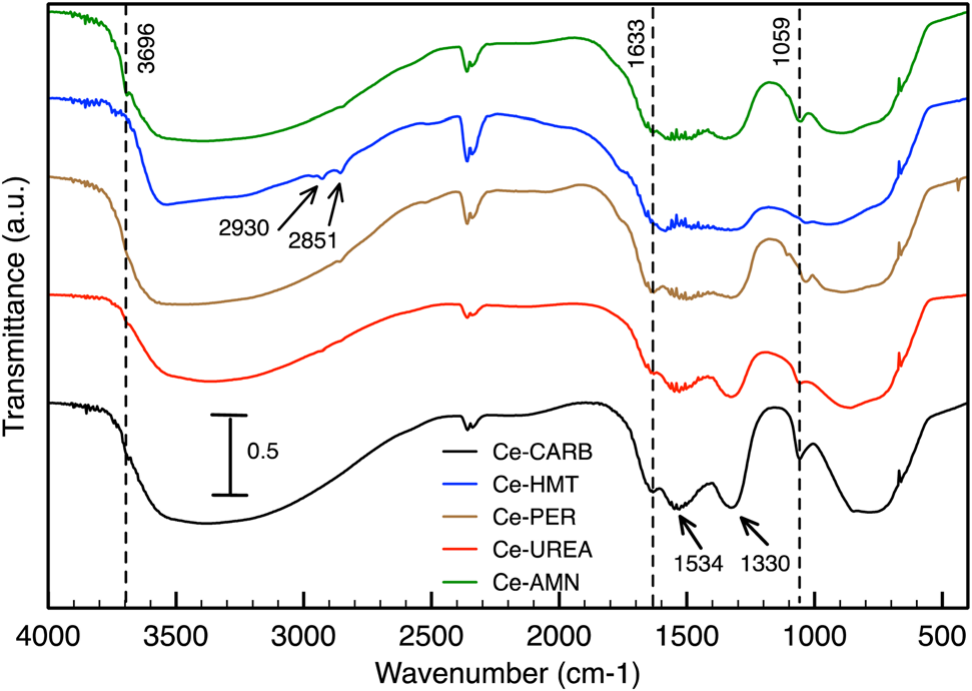
DRIFT spectra of prepared ceria samples.

The DRIFT spectra of prepared ceria samples are presented in Fig. 5. The characteristic bands corresponding to –OH stretching, attributed to physically adsorbed water or various types of surface –OH groups^13,77^ are localized in the 3700–2700 cm^−1^ range. These bands correlate well with those at 1633 cm^−1^ and 1059 cm^−1^, which are associated with molecular H_2_O (H–O–H) bending vibration.^78,79^ According to Hadjiivanov,^80^ the low-intensity band around 3696 cm^−1^ can be assigned to the type I hydroxyls. The spectrum of Ce-CARB exhibits characteristic stretching vibrations (at 1330 and 1534 cm^−1^) of bicarbonate-like species (O–C–O),^81,82^ which can be correlated with the relative amount of residual carbonates.^83^ In the Ce-HMT spectrum, weak bands at 2930 and 2851 cm^−1^ correspond to the CH_2_ stretching vibration originating from the HMT residues.^84^ Bands associated with carbonate-like species on the ceria surface, resulting from interactions between atmospheric CO_2_ and cerium cations, are observed in the 1500–1000 cm^−1^ range.^83,85^ Additionally, low-intensity bands corresponding to the stretching vibrations of Ce–O bonds are localized in the low-frequency range of 850–500 cm^−1^.

**Fig. 6:**
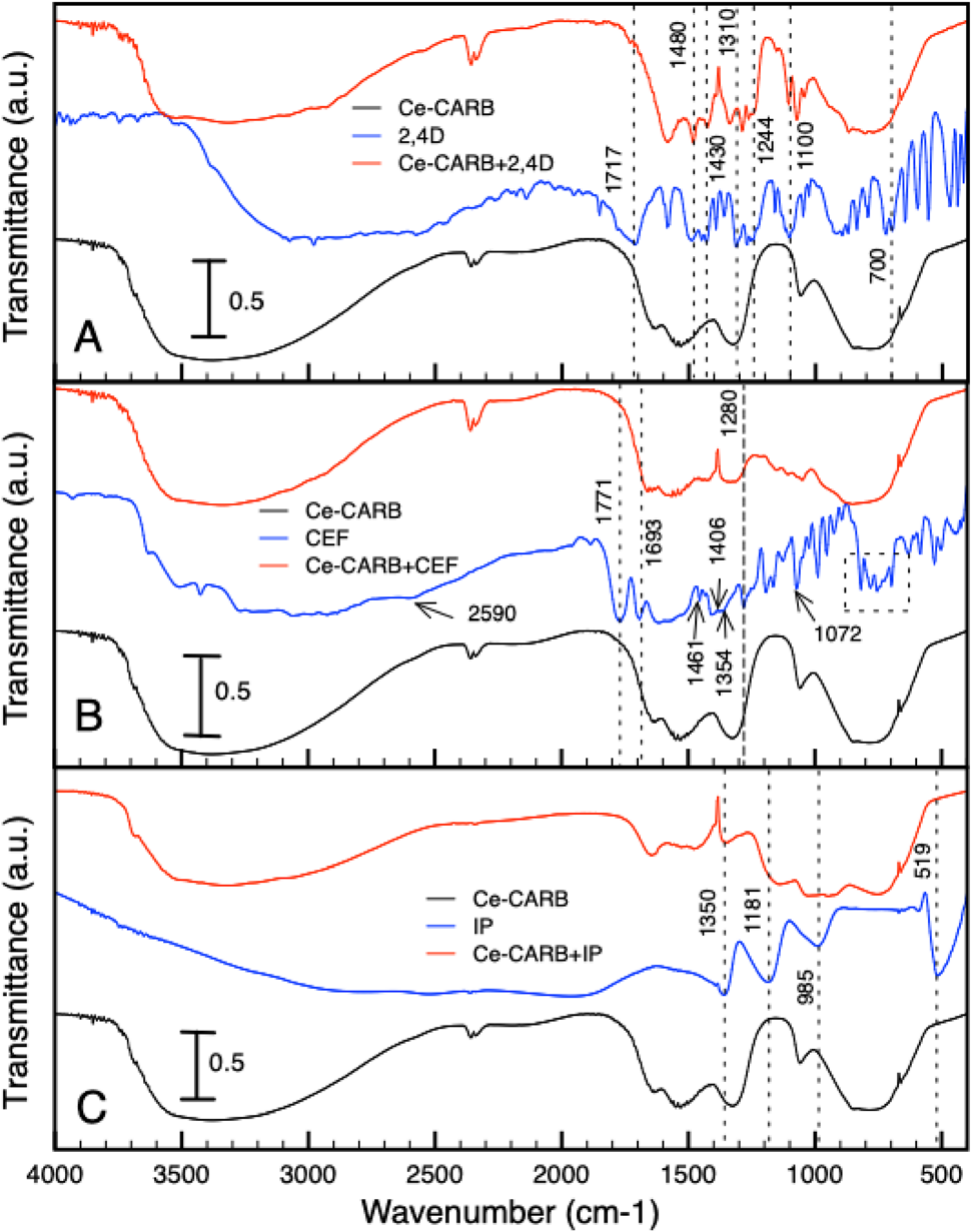
DRIFT spectra of Ce-CARB (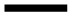), pure pollutant (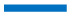), and Ce-CARB after pollutant adsorption (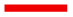) for 2,4D (A), CEF (B), and IP (C).

The ceria surface interaction with 2,4D, CEF, and IP was evaluated from DRIFT spectra in Fig. 6A–C. Furthermore, only the data for Ce-CARB are presented; similar data were obtained for all ceria samples. The DRIFT spectra of Ce-CARB before (black line), after pollutant adsorption (red line), and the pure forms of 2,4D, CEF, and IP (blue line). The spectra of 2,4D (Fig. 6A) show characteristic bands at 1480 and 1430 cm^−1^, corresponding to the C=C vibrations of the aromatic ring and the –CH_2_ vibrations of alkenes, respectively. The symmetric and antisymmetric vibrations of C–O–C are observed at 1310 and 1100 cm^−1^. A band due to O–H deformation combined with C–O stretching vibrations appears at 1244 cm^−1^.^31,64^ Additionally, the peak at 700 cm^−1^ indicates C–Cl stretching.^64,86^ The band at 1717 cm^−1^ corresponds to the –C=O stretching vibrations of the carboxyl group,^31^ which overlaps with the C–O stretching vibrations at 1233 cm^−1^.^86^ After the adsorption of 2,4D onto Ce-CARB, some of the original bands of 2,4D are suppressed or disappear, while new bands are formed. Specifically, new bands were observed at 1584, 1480, 1430, 1288, 1153, 1100, 1042, and 877 cm^−1^, whereas bands at 1636, 1542, and 1332 cm^−1^ disappeared. Additionally, some peaks shift due to electron donation interactions between 2,4D and ceria samples.^64^ These bands (1332, 1430, and 1717 cm^−1^) can be associated with the C=O vibration of the 2,4D anion. The bands at 1430 and 1480 cm^−1^ correspond to the C=C vibrations of the aromatic ring.^86^ Furthermore, the bands at 1584 and 1430 cm^−1^ are attributed to the ring deformation vibration of β-CH + γ-CH (ring) bending and CH_2_ bending combined with C–C and C–O stretching, respectively.^87^

The spectra of CEF, Ce-CARB after CEF adsorption, and the Ce-CARB sample are presented in Fig. 6B. The characteristic bands of the CEF molecule, corresponding to the four-membered lactam carbonyl and the secondary amide carbonyl group, are located at 1771 cm^−1^ and 1693 cm^−1^, respectively.^88,89^ The bands at 1461, 1406, and 1354 cm^−1^ correspond to C–H bending vibrations. The band at 1280 cm^−1^ is attributed to C–N stretching vibrations, while the band at 1072 cm^−1^ corresponds to C–O stretching vibrations. The characteristic bands of the monosubstituted phenyl groups are observed at 745 and 696 cm^−1^.^89^ A weak band around 2590 cm^−1^ is associated with the stretching vibration of the S–H bond.^32^ After adsorption, certain new bands appeared (*e.g.*, at 1413 and 1350 cm^−1^), while others disappeared or were suppressed (*e.g.*, bands around 1600 cm^−1^ and 1000–600 cm^−1^ region), suggesting an interaction between the quaternary ammonium salt-like primary amine group of CEF and the surface hydroxyl groups of the ceria sample, as well as hydrogen bonding involving the amino, thiol, and carboxylate groups of CEF. These findings align with the data^89–91^ and indicate the chemical stability of CEF after adsorption onto the ceria surface.

**Fig. 7:**
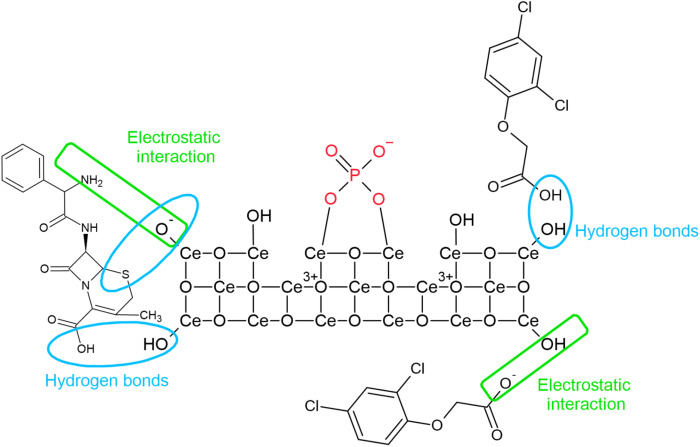
Proposed interaction mechanism of individual pollutants (IP, CEF and 2,4D) with ceria surface.

The spectra of IP, Ce-CARB after IP adsorption, and the Ce-CARB sample are shown in Fig. 6C. After IP adsorption (red line), the bands in the 1600–1200 cm^−1^ range associated with bicarbonate-like species are suppressed. This is likely due to the interaction of phosphate with residual carbonates,^81,82^ which can play a role in the adsorption and catalytic activity of ceria.^92^ However, according to information in ref. 93 and 94, the intensity of bands may be related to the phosphorus amount loaded on the surface. The strong band at 519 and 985 cm^−1^ corresponds to the out-of-plane P–OH bending vibration and PO–H bending vibration, respectively.^95^ After the adsorption of inorganic phosphates, the new bands at 1134 and 1031 cm^−1^ appeared, which were assigned to *ν*_3_ triply degenerate asymmetric stretching mode of P–O groups.^96^ The bands in the range 1200–400 cm^−1^ are connected with various vibrations of PO_4_^3−^ groups suggesting the creation of insoluble CePO_4_.^49,97^ The weak band at 1634 cm^−1^ could probably be ascribed to the O=P–OH deformation vibration, and these bands are shifted probably due to the decrease in the bending force of O=P–OH deformation on ceria. The band at 1350 and 1181 cm^−1^ corresponds to the P=O stretching vibration.^95^ According to FTIR data, the suggested interaction mechanism with the ceria surface is presented in Fig. 7. The most predominant interactions are electrostatic and hydrogen bond with ceria surface –OH groups and the functional groups (*i.e.*, –NH_2_, –OH) or some atoms (like S, O) in the pollutants.

## 4. Conclusions

In this study, a set of nanocrystalline ceria samples was prepared using various wet chemical methods to evaluate their adsorption capacity for IP, 2,4D, and CEF. The ceria samples were characterized using advanced instrumental techniques including BET, XRD, XRF, and FTIR, as well as through classical analytical chemistry methods. The specific interactions of IP, 2,4D, and CEF were investigated and confirmed using FTIR. The adsorption behavior of CEF, 2,4D, and IP on ceria samples is influenced by the physicochemical properties of ceria, including specific surface area, pore volume, crystallite size, and surface charge. The negative Δ*G*° values confirm that the adsorption process is spontaneous and feasible for all samples and pollutants, requiring no external energy input, predominantly governed by physisorption. The adsorption of IP aligns with the Freundlich model, indicating a predominantly physical process, whereas the mechanisms of IP adsorption involve Ce^3+^ sites and the formation of insoluble CePO_4_ species. The presence of phosphorus loaded on ceria (after IP adsorption) was confirmed by FTIR, XRF, and XRD. The adsorption of CEF and 2,4D is primarily governed by SSA, CCS, and pore volume, with Ce-AMN demonstrating the highest capacity due to its optimal properties. The adsorption process can be well fitted with the Langmuir–Freundlich model. Whereas the adsorption kinetics for CEF, IP and 2,4D align with pseudo-second-order kinetics suggesting that the process is controlled by chemisorption. These findings highlight the effectiveness of ceria-based materials in pollutant removal due to their surface heterogeneity and specific physicochemical characteristics. While this study demonstrates the significant potential of nanocrystalline ceria, further investigations are required to ascertain its practical viability for water treatment. Future work should therefore prioritize evaluating the adsorbent's efficacy and selectivity in complex environmental samples containing a variety of competing inorganic and organic species.

## Author contributions

The manuscript was written through the contributions of all authors. All authors have given approval to the final version of the manuscript.

## Conflicts of interest

There are no conflicts to declare.

## Supplementary Material

RA-015-D5RA05301C-s001

## Data Availability

The raw data required to reproduce the above findings are available to download from: https://doi.org/10.5281/zenodo.15465200. Supplementary information: description of sample characterization methods, SEM images of the prepared ceria sample. results of acid–base sample characterization with corresponding figures. separation factor for adsorption isotherms. XPS analysis, XRF and XRD results. See DOI: https://doi.org/10.1039/d5ra05301c.
