# Supplementary material for: Physicochemical aspects of adsorption efficiency of nanocrystalline ceria toward antibiotics, herbicides, and inorganic phosphates
Source: RSC Adv. 2025 Oct 14;15(45):38391–405. doi: 10.1039/d5ra05301c (PMC12519946; doi:10.1039/d5ra05301c)
Supplement: RA-015-D5RA05301C-s001 [file RA-015-D5RA05301C-s001.pdf]

Supplementary Information (SI) for RSC Advances.
This journal is © The Royal Society of Chemistry 2025

Supporting information

Physicochemical aspects of adsorption efficiency of nanocrystalline ceria toward antibiotics, herbicides, and inorganic phosphates

Jakub Ederer[1*], Luboš Vrtoch[2], Petr Ryšánek[2], Matouš Bárta[1], Viktorie Neubertová[2], Zdeňka Kolská[2]

[1]Faculty of Environment, Jan Evangelista Purkyně University, Pasterurova 3632/15, 400 96 Ústí nad Labem, Czech Republic

[2]Faculty of Science, Jan Evangelista Purkyně University, Pasteurova 3632/15, 400 96, Ústí nad Labem, Czech Republic

[*]**Corresponding author**: Tel: +420-475-284-111; Fax: +420-475-284-158.

E-mail: jakub.ederer@ujep.cz (Jakub Ederer).

Characterization of samples

All samples were measured three times with an experimental error of 5 %. Five-point Brunauer–Emmett–Teller (BET) analysis and micropore analyses were applied to obtain total surface area, and the Density Functional Theory (DFT) model was used to determine pore volume from the nitrogen adsorption/desorption isotherm.

Samples were prepared by dispersing the powders in methanol and drop-casting the suspension onto silicon wafers mounted on SEM stubs with conductive carbon adhesive tape. No conductive coating was applied prior to analysis. The measurements were performed in high vacuum mode using a secondary electron detector at an accelerating voltage of 30 kV.

Surface acid-base characterization

Briefly, the titration was performed on an automatic titrator controlled by a PC (794 Basic Titrino, Metrohm, Switzerland) with a potentiometric endpoint determination. A 500 mg ceria sample was added to 50 mL of NaCl (0.1 M) along with 3 mL of standardized HCl solution (0.1 M in 0.1 M NaCl), and the suspension was mixed and bubbled with nitrogen for 15 minutes. Furthermore, the suspension was titrated with standardized NaOH (0.1 M in 0.1 M NaCl) under continuous stirring and a nitrogen atmosphere until a pH of 11.5 was achieved. The titrant rate was 0.1 mL/min in 0.05 mL aliquots.

Electrophoretic light scattering (ELS) was applied to determine the zeta potential. The system was integrated with a Metrohm automatic titrator featuring an 867 pH module and 846 dosing interface, all operated via Kalliope™ software. The ceria dispersions were prepared in deionized water, and each measurement used two aliquots of the same sample. One aliquot was titrated from its native pH to pH 12 using 0.1 M NaOH, while the other was titrated to pH 2 using 0.1 M HCl, both with 0.2 pH unit increments. Results from both titrations were consolidated into a single plot. Zeta potential values were computed using the Smoluchowski approximation, offering insight into surface charge behavior and chemical characteristics relevant to the colloidal stability and interaction potential of the ceria particles.

Dynamic light scattering (DLS) was employed on the same instrument to determine the hydrodynamic diameter and size distribution of the ceria nanoparticle suspensions. Measurements were conducted at a constant temperature of 25 °C, using a 40 mW red semiconductor laser (658 nm) and a fixed backscattering detection angle of 175°.

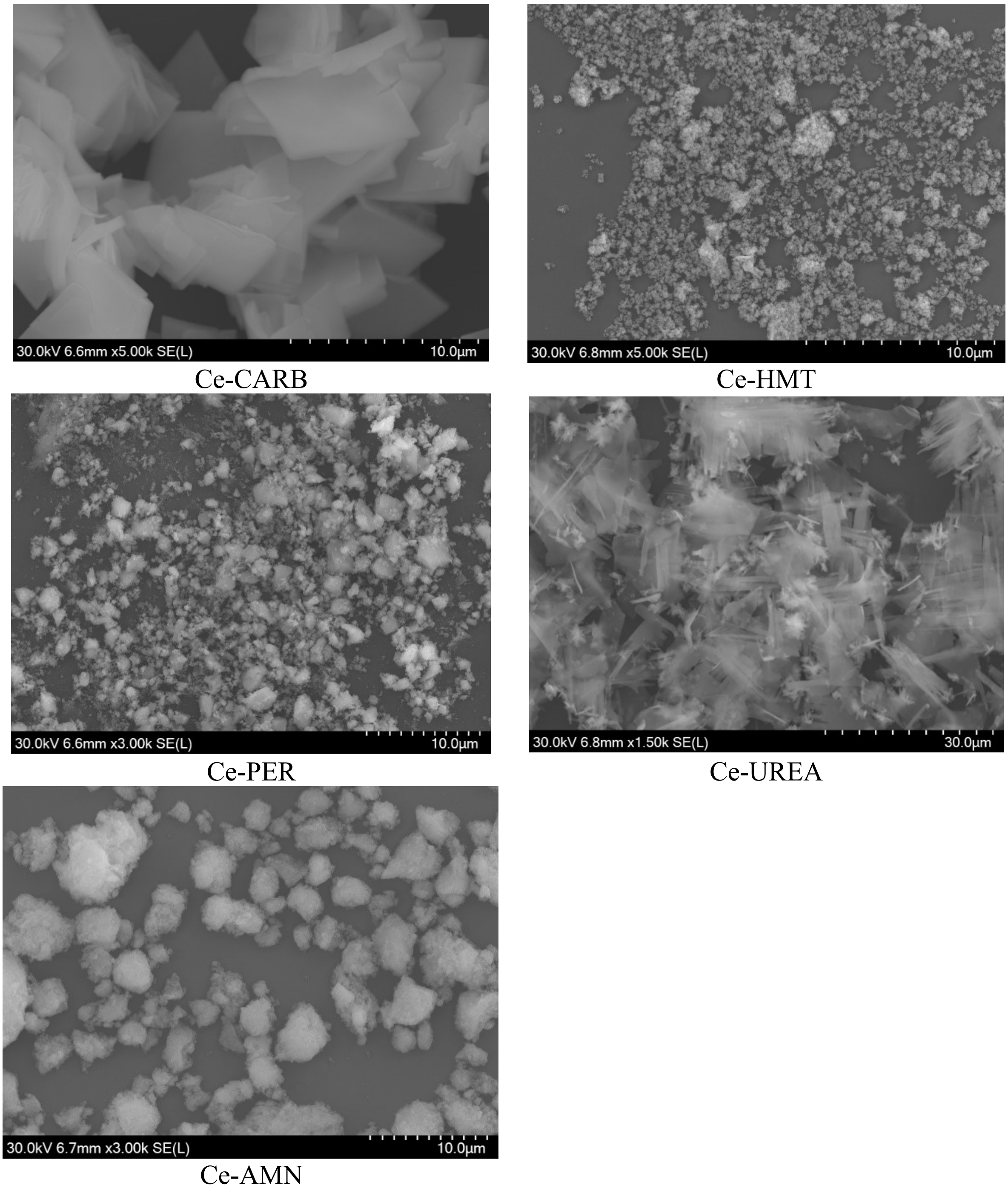

Fig. S1 SEM nanostructure of prepared ceria samples.

Results and discussion

Surface acid-base characteristics

The TOTH and 1st derivation of titration curves are presented in Fig. S2 A, B. The data summarized in Table 2 show significant differences in the number of surface hydroxyl groups for the various synthesis procedures. In contrast, the pH(PZC) remains similar, except for the Ce-HMT sample. The Ce-HMT higher pH(PZC) value could be associated with the remaining HMT residues. The calculated number of hydroxyl groups and pH(PZC) values nicely correlated with the data published elsewhere [1].

Zeta potential determinations were used to evaluate the surface charge and stability of ceria samples in aqueous solution. Fig. S3 A shows the ceria sample's zeta potential as a function of pH, and the calculated data are listed in Table 2. The isoelectric point (IEP) of ceria samples was evaluated from the plot in Fig. S3 A, and the values are summarized in Table 2. The IEP values are consistent with previously reported values for ceria samples using different synthesis methods [2–4]. Variation in zeta potential and IEP can be attributed to differences in the crystalline structure [4] or the ceria synthesis method. Additionally, the degree of surface hydration is linked to the surface -OH groups, which can acquire positive or negative charges depending on the pH [4].

The measured data (Table 2) indicate that the particles are positively charged at pH<5.21 due to the protonation of hydroxyl surface groups (Eq. 1). Based on their IEP values, the samples are ordered as follows: Ce-AMN<Ce-CARB<Ce-UREA<Ce-HMT<Ce-PER. Above pH=5.21, the particles become negatively charged due to deprotonation (Eq. 2).

$$ceria - OH + H^{+} \leftrightarrow ceria - OH_2^{+} \quad (1)$$

$$ceria - OH \leftrightarrow ceria - O^{-} + H^{+} \quad (2)$$

This behavior is consistent with that of metal oxide nanoparticles reported in the literature [5,6]. At pH levels above 9.0 or below 4.0, the particles acquire a strong anionic or cationic charge, respectively, and demonstrate high stability, as reflected in their zeta potential values ($|\zeta| > 30$ mV) [7].

The hydrodynamic particle size distribution of ceria samples measured using dynamic light scattering (DLS) is presented in Fig. S3 B. The prepared ceria particles are well dispersed in water, according to DLS results measured at native pH. The mean particle sizes for the Ce-CARB, Ce-HMT, Ce-PER, Ce-UREA, and Ce-AMN samples were 1667, 2021, 754, 1084, and 1261 nm, respectively. The DLS data are inconsistent with the results obtained from SEM and XRD observations, likely due to the more favorable formation of larger agglomerates in an aqueous environment through the surface charge of ceria particles. The calcined samples exhibit

larger particle sizes due to sintering and the formation of larger agglomerates. In contrast, the samples that were only dried (Ce-AMN, Ce-PER, Ce-HMT) demonstrate smaller particle or agglomerate sizes, consistent with the theoretical expectations. Table 2 presents the average particle size (diameter) and the polydispersity index (PDI). A PDI value below 0.30 indicates a uniform particle size distribution and good suspension stability of the ceria samples in water at their native pH [8]. This suggests that most particles are relatively similar in size, although minor variations exist within the sample. However, the Ce-CARB sample shows inconsistent results, making it difficult to determine if it exhibits a uniform particle size distribution due to its PDI being close to 0.30.

The main differences between pH(IEP) and pH(PZC) were discussed and described in [9]. Briefly, these values should be same if no other reaction or adsorption process is present [10]. Differences between pH(IEP) and pH(PZC) and the shift in pH(IEP) values are primarily caused by the specific adsorption of cations or anions from the electrolyte used during zeta potential measurement [11]. In contrast, acid-base titration is based on the transfer of OH^- and H_3O^+ ions between the ionizable surface groups of ceria. Zeta potential (electrophoretic mobility), however, corresponds to the external surface charge potential. Variations between pH(PZC) and pH(IEP) indicate greater heterogeneity in prepared ceria samples and can be attributed to the specific adsorption of cations or anions from the background electrolyte, as well as differences in prepared samples, such as purity, sample preparation history, experimental protocols or crystallinity of the sample [11].

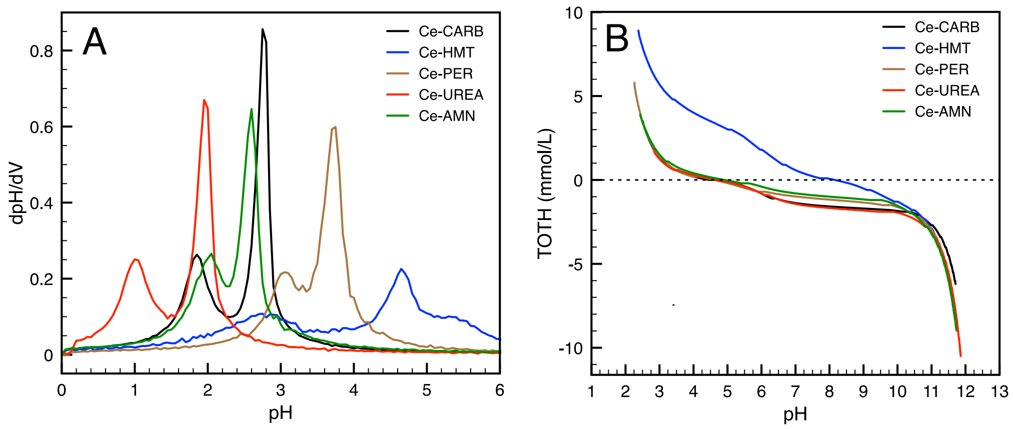

Fig. S2 (A) 1st derivation of titration curves of the prepared samples with two distinct equivalent points (EP1 and EP2). (B) Curves corresponding to the total concentration of protons consumed in the titration process (TOTH).

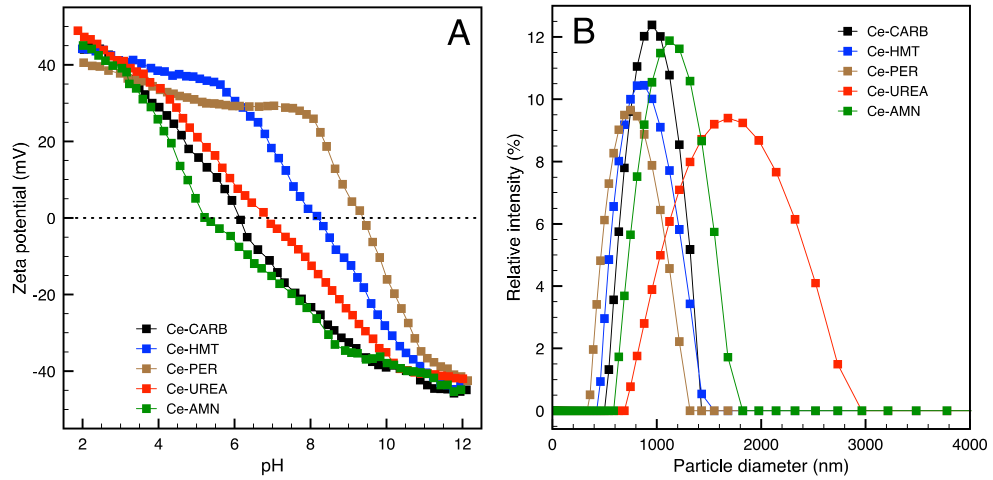

Fig. S3 Zeta potential (A) and DLS particle size distribution (B) measurements for prepared ceria samples.

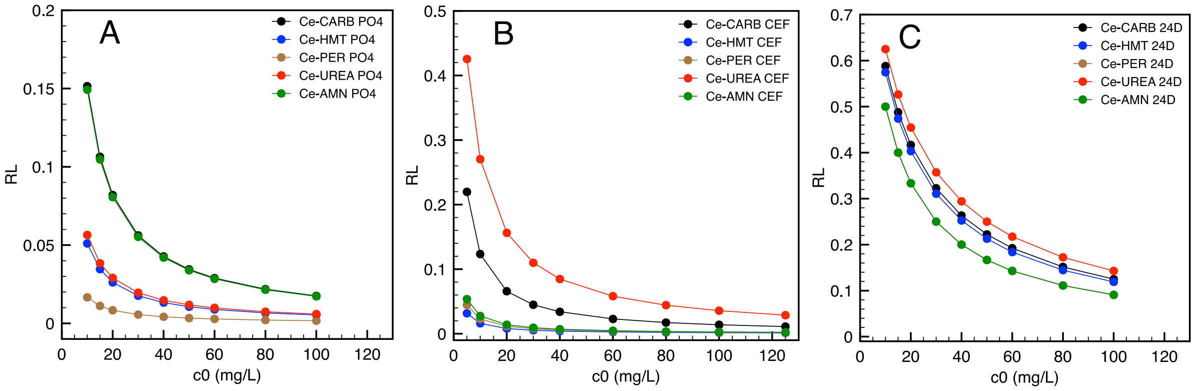

Fig. S4 Dependence of separation factor (R_L) on the initial concentration (c_0) for inorganic phosphate (A), cephalexin (B), and 2,4D (C).

Table S1. Elemental surface composition of samples obtained from XPS [1].

Sample	Atomic concentration					
	Ce^{3+} [%]	Ce^{4+} [%]	Ce [at%]	O_{Ce4+} [%]	O_{Ce3+} [%]	O_{OH} [%]
Ce-AMN	7	93	22.4	85.3	9.4	5.3
Ce-CARB	7	93	26.8	92.8	3.1	4.1
Ce-HMT	16	84	23.3	62.6	18.4	19.0
Ce-PER	11	89	20.5	54.3	29.0	16.7
Ce-UREA	12	88	26.7	95.1	0.5	4.4

Table S2. XRF analysis of ceria samples before and after IP adsorption.

Sample/ Component	Ce-CARB		Ce-UREA		Ce-PER		Ce-HMT		Ce-AMN	
	clean	with IP	clean	with IP	clean	with IP	clean	with IP	clean	with IP
	mass%									
CeO_2	99.9	98.5	99.9	98.6	98.7	97.2	99.9	97.4	99.8	99.1
P_2O_5	0.0	1.04	0.0	0.872	0.0	1.45	0.0	1.81	0.0254	0.818
F	0.0	0.0	0.0	0.0	0.0	0.0	0.0	0.0	0.0	0.0
SiO_2	0.0299	0.0	0.0	0.0	0.987	0.757	0.0485	0.0682	0.0661	0.0
SO_3	0.0922	0.0525	0.0955	0.0881	0.0939	0.0823	0.0419	0.0384	0.0511	0.0377
K_2O	0.0	0.362	0.0	0.304	0.0	0.428	0.0	0.634	0.0	0.0920
Al_2O_3	0.0	0.0	0.0	0.0	0.0821	0.0931	0.0	0.0	0.0451	0.0
Cl	0.0	0.0	0.0	0.0	0.0468	0.0	0.0	0.0	0.0	0.0
MnO	0.0	0.0	0.0	0.0	0.0926	0.0	0.0	0.0	0.0	0.0
Fe_2O_3	0.0	0.0	0.0	0.0	0.0	0.0	0.0	0.0778	0.0	0.0

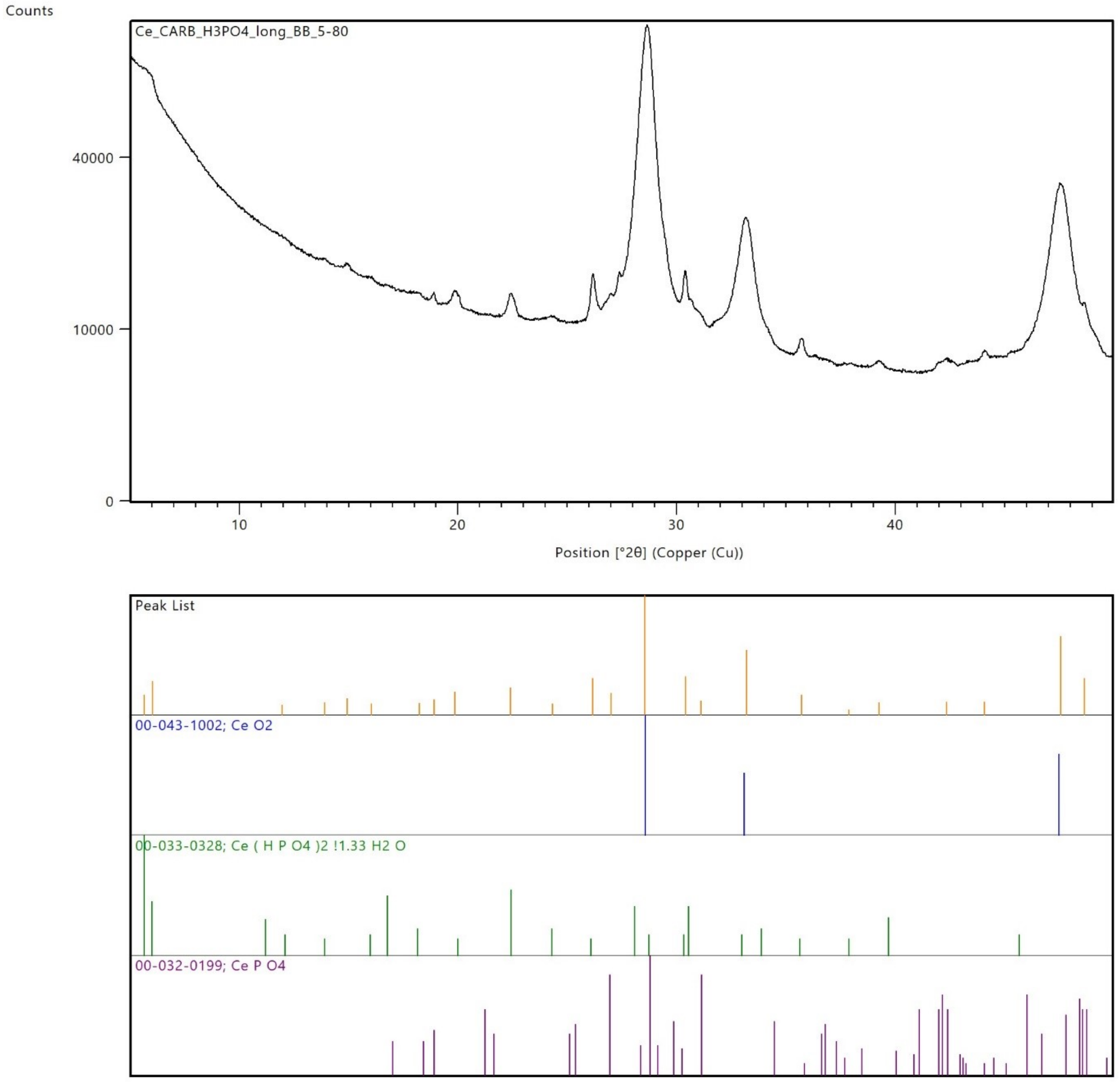

Fig. S5 Diffraction pattern of Ce-CARB after adsorption of IP and comparison with XRD pattern with databases.

References

1 J. Henych, M. Šťastný, J. Ederer, Z. Němečková, A. Pogorzelska, J. Tolasz, M. Kormunda, P. Ryšánek, B. Bażanów, D. Stygar, K. Mazanec and P. Janoš, *Environ. Sci. Nano*, 2022, **9**, 3485–3501.

2 M. Brigante and P. C. Schulz, *Chem. Eng. J.*, 2012, **191**, 563–570.

3 J. Henych, M. Šťastný, Z. Němečková, M. Kormunda, Z. Šanderová, Z. Žmudová, P. Ryšánek, Š. Stehlík, J. Ederer, M. Liegertová, J. Trögl and P. Janoš, *ACS Appl. Nano Mater.*, 2022, **5**, 17956–17968.

4 J. J. Gulicovski, I. Bračko and S. K. Milonjić, *Mater. Chem. Phys.*, 2014, **148**, 868–873.

5 N. Wang, C. Hsu, L. Zhu, S. Tseng and J.-P. Hsu, *J. Colloid Interface Sci.*, 2013, **407**, 22–28.

6 C. H. Veloso, L. O. Filippov, I. V. Filippova, S. Ouvrard and A. C. Araujo, *J. Mater. Res. Technol.*, 2020, **9**, 779–788.

7 A. Kumar and C. K. Dixit, in *Advances in Nanomedicine for the Delivery of Therapeutic Nucleic Acids*, Elsevier, 2017, pp. 43–58.

8 S. Gurunathan, J. Raman, S. N. Abd Malek, P. A. John and S. Vikineswary, *Int. J. Nanomedicine*, 2013, **8**, 4399–4413.

9 J. Ederer, P. Ecorchard, M. Š. Slušná, J. Tolasz, D. Smržová, S. Lupínková and P. Janoš, *Adsorpt. Sci. Technol.*, 2022, **2022**, 1–16.

10 M. Alvarez-Silva, M. Mirnezami, A. Uribe-Salas and J. A. Finch, *Can. Metall. Q.*, 2010, **49**, 405–410.

11 T. Mahmood, M. T. Saddique, A. Naeem, P. Westerhoff, S. Mustafa and A. Alum, *Ind. Eng. Chem. Res.*, 2011, **50**, 10017–10023.
